# 799. A Pseudo-Outbreak of *Pseudomonas fluorescens* Infections

**DOI:** 10.1093/ofid/ofab466.995

**Published:** 2021-12-04

**Authors:** Tariq Jaber, Vikram Saini, Laura Morris, James D Como, Nitin Bhanot, Zaw Min

**Affiliations:** 1 Allegheny General Hospital, Pittsburgh, Pennsylvania; 2 Allegheny Health Network, Pittsburgh, Pennsylvania; 3 Infectious Disease, Allegheny General Hospital, pittsburgh, Pennsylvania

## Abstract

**Background:**

*Pseudomonas fluorescens* is a water-borne pathogen that has been associated with outbreaks from transfusion of contaminated blood products or medical equipment. Our institution had a cluster of cultures that grew an uncommonly encountered microbe *P. fluorescens* within a period of one week. This prompted an internal investigation. We summarize the investigational process that led to the resolution of this pseudo-outbreak.

**Methods:**

We conducted a retrospective chart review of surgical and non-surgical patients with cultures positive for *P. fluorescens* from July 2^nd^ to July 8^th^ 2020. Baseline patient characteristics, clinical course, laboratory data, use of blood-associated products, and microbiology cultures were analyzed.

**Results:**

Eight patients were identified with positive tissue cultures for *P. fluorescens*. Among those, 5 specimens (62.5%) were from osteoarticular sites (1 prosthetic hip, 1 prosthetic knee, 1 right foot, 1 sternum, and 1 vertebral source). One culture (12.5%) was obtained from a sacral soft tissue wound. Two tissue specimens (25%) were collected from respiratory sites (1 lung tissue and 1 bronchoalveolar lavage). No association with specific surgical personnel or operating room was identified. During routine specimen processing, a small amount of sterile normal saline is added to the conical grinder prior to culture preparation. It was discovered that a non-sterile normal saline had been inadvertently utilized during that step. These eight tissue specimens were subsequently reprocessed with sterile solution; *P. fluorescens* was not re-isolated. Specimen processing protocols were reinforced. Adjustment of antimicrobial therapy was made accordingly without reported subsequent adverse clinical outcomes.

**Conclusion:**

A multi-faceted team approach in collaboration with Infection Prevention, Infectious Diseases, Surgery, operating room personnel, and Microbiology identified an unintended breakdown in sterile laboratory protocols which resulted in a cluster of falsely positive cultures. An increased incidence of infection with an uncommon pathogen initiated a prompt investigation that resulted in the identification of a pseudo-outbreak event.

**Disclosures:**

**All Authors**: No reported disclosures

